# Inference of Biological Pathway from Gene Expression Profiles by Time Delay Boolean Networks

**DOI:** 10.1371/journal.pone.0042095

**Published:** 2012-08-31

**Authors:** Tung-Hung Chueh, Henry Horng-Shing Lu

**Affiliations:** 1 Green Energy and Environment Research Laboratories, Industrial Technology Research Institute, Chutung, Hsinchu, Taiwan, Republic of China; 2 Institute of Statistics, National Chiao Tung University, Hsinchu, Taiwan, Republic of China; Queen’s University Belfast, United Kingdom

## Abstract

One great challenge of genomic research is to efficiently and accurately identify complex gene regulatory networks. The development of high-throughput technologies provides numerous experimental data such as DNA sequences, protein sequence, and RNA expression profiles makes it possible to study interactions and regulations among genes or other substance in an organism. However, it is crucial to make inference of genetic regulatory networks from gene expression profiles and protein interaction data for systems biology. This study will develop a new approach to reconstruct time delay Boolean networks as a tool for exploring biological pathways. In the inference strategy, we will compare all pairs of input genes in those basic relationships by their corresponding 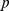-scores for every output gene. Then, we will combine those consistent relationships to reveal the most probable relationship and reconstruct the genetic network. Specifically, we will prove that 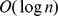 state transition pairs are sufficient and necessary to reconstruct the time delay Boolean network of 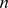 nodes with high accuracy if the number of input genes to each gene is bounded. We also have implemented this method on simulated and empirical yeast gene expression data sets. The test results show that this proposed method is extensible for realistic networks.

## Introduction

In order to understand complex biological networks and pathways, we need to investigate global structures instead of individual behaviors since there are interactions and associations between genes. Due to the invention of high throughput technology, genome-wide expression profiles can be measured simultaneously [1]. However, it is still a great challenge to identify complex biological networks from genome-wide data because the number of gene interactions is huge [2]. In recent years, there has been a significant progress in research concerning genetic network models and network reconstruction problems.

Clustering and dimension reduction are important methods for grouping genes that have similar expression profiles [3], [4]. In the framework of clustering, it is important to define the degree of similarity between genes. By the method of clustering, we can group genes that have similar expressions. However, we still cannot find the causal relationship between genes. Hence, apart from the relationship of similarity, we will also have to consider another causal relationship between genes.

There have been many methods proposed in the literature to tackle the problem of genetic regulatory network reconstruction. For instance, the steady state approach have been used to model gene regulatory networks [5]. In addition, the Bayesian network model is an important technique that has been studied extensively in the past two decades [6]–[11]. A Bayesian network is a directed acyclic graph (DAG) comprised of two components. The first component is comprised of nodes that correspond to a set of variables and a set of directed edges between variables with Markov properties. The second component describes a conditional distribution for each variable given its parents in the graph. Recently, Bayesian network models have been applied to analyze microarray expression and biological data [12]–[15]. However, Bayesian network algorithms have limitations when dealing with large-scale gene regulatory networks because of their complex modeling structure [16]. Although algorithms for reconstructing Bayesian networks have already been developed [17], [18], the algorithms’ computational costs remain a concern for the searching of all potential network structures on the genome-wide expression data.

Therefore, we are considering a simpler model: Boolean networks, which have been studied extensively in a variety of contexts. Boolean networks [19], [20] can effectively explain the dynamic behaviors of living systems. Moreover, for large-scale gene regulatory networks, Kim et al. [21] have used Boolean network with chi-square test on the yeast cell cycle microarray gene expression data sets. The chaos and attractors of Boolean network are also discussed widely from the aspect of power spectrum [22]–[24]. Recently, Boolean network also have been used as a discrete model for the *lac* operon [25].

**Figure 1:**
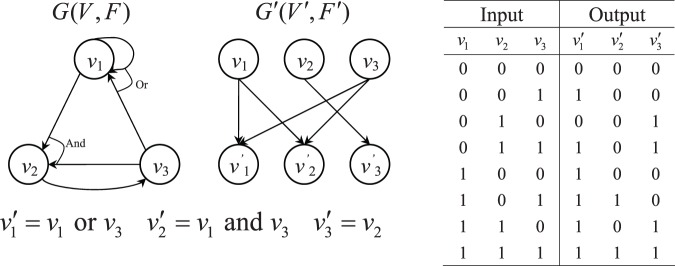
Boolean network *G*(*V,F*), wiring diagram *G′*(*V′,F′*) and its input/output.

Boolean networks were originally introduced by Kauffman, and received attention in the studies of gene regulatory networks because of their simple structures [26]. In a Boolean network model, nodes represent the gene expression states. The status of a gene is quantized to one of the two states: on or off, representing a gene as active or inactive respectively. The wiring of rules between nodes in the graph represents a functional link between genes and determines the expressions of target genes after giving a series of input genes. Under the structure of Boolean networks, the target gene is determined by a set of genes with specific rules. For each gene, if the indegree (i.e., the number of input genes to each gene) is bounded by a constant 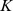, only 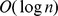 pairs of state transition are necessary and sufficient to reconstruct the original network with 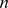 nodes [27], [28]. However, Boolean networks have been criticized for their deterministic nature. The assumption that every affected gene would be expressed immediately at the next time step may be unsound.

**Figure 2:**
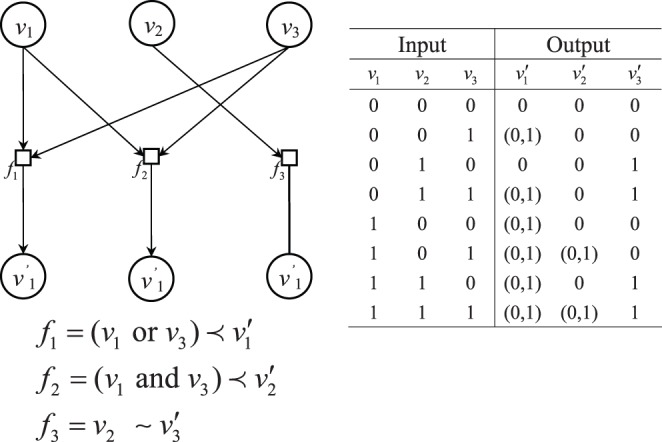
One example of time delay Boolean network and its input/output.

Another point of view of constructing genetic network is to focus on the indication the pairwise relationships between genes. Most of the works is to find the gene-pairs with similarity relationship [29]–[33]. The similarity of a gene-pair represents the two genes with the same expression or opposite expression. In 2005, Li and Lu proposed directed acyclic Boolean network and the statistical reconstruction method of SPAN to infer the pair wise relations of every element [34]. The proposed method can reconstruct Boolean networks from noisy array data by assigning an s-p-score for every pair of genes. In the study, they proposed another relationship between two genes: relationship of prerequisite under the Boolean network model. If gene 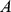 is a prerequisite for gene 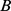, then the “on” status of gene 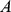 is necessary for the “on” status of gene 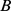. Boolean implication network, with the similar aspect, investigated all Boolean implication between pairs of gene for large scale genome microarray datasets [35]. Following the model, Wang et al.[36] proposed a two step counting approach for constructing biological pathways with Boolean network. However, most of these methods only consider pair wise relationship in order to decrease the time complexity. Therefore, if the structure of network is a combination of a set of genes to affect another gene, the algorithms will lose some information and rules in the genetic network reconstruction.

**Table 1:** Count and probabilities table for 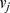, 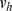 and 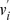 assuming no misclassification error.

*v′_i_/v_j_v_h_*	00	01	10	11	*v′_i_/v_j_v_h_*	00	01	10	11
0	*m*_000_	*m*_010_	*m*_100_	*m*_110_	0	*q*_000_	*q*_010_	*q*_100_	*q*_110_
1	*m*_001_	*m*_011_	*m*_101_	*m*_111_	1	*q*_001_	*q*_011_	*q*_101_	*q*_111_

In this study, we will consider a much more generalized model by combining the structure of the above two models. If a Boolean function with one or several genes is a prerequisite for a target gene, then the induction of the Boolean function with input genes is necessary for the expression of the target gene. Hence, the target will be influenced by the Boolean function with several input genes. However, the induction of the Boolean function may not activate the target gene immediately, but at a future time. Therefore, the target gene may not have been influenced right now and we will treat these relationships as time delay affection. In this study, we will infuse these additional relationships for more generalized systems.

**Table 2:** Count profiles for the basic eight relationships without misclassification error.

(*v_j_* or *v_h_*) 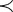 *v′_i_*					(*v_j_* or *v_h_*) 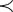 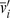				
*v′_i_/v_j_v_h_*	00	01	10	11	*v′_i_/v_j_v_h_*	00	01	10	11
0	+	+	+	+	0	0	+	+	+
1	0	+	+	+	1	+	+	+	+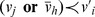					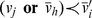				
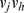 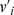 /	00	01	10	11	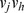 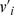 /	00	01	10	11
0	+	+	+	+	0	+	0	+	+
1	+	0	+	+	1	+	+	+	+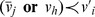					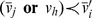				
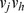 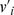 /	00	01	10	11	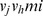 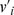 /	00	01	10	11
0	+	+	+	+	0	+	+	0	+
1	+	+	0	+	1	+	+	+	+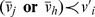					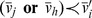				
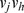 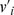 /	00	01	10	11	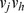 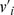 /	00	01	10	11
0	+	+	+	+	0	+	+	+	0
1	+	+	+	0	1	+	+	+	+

### Boolean Network

Boolean networks were introduced by Kauffman (1969) forty years ago to represent genetic regulatory networks. First, we will review the definition of a Boolean network. A Boolean network 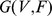 is a directed graph consisting of two components: a set of nodes 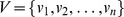 that corresponds to genes, and a list of Boolean functions 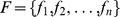 that corresponds to the rule of interaction and combination of several genes. For every node 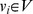, its expression is simplified to two levels: on and off, representing a gene as active or inactive. For every Boolean function 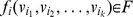, 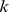 specified input nodes 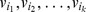 are assigned to the node 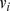 in the graph and represent the rules of regulatory mechanisms between genes. The expression of a gene is determined by the expression of the gene directly affecting it with a Boolean function. Therefore, the state of each node 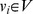 is determined by the Boolean function 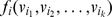.

For each node 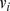, the gene expression state at time 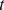 is assumed to take either 0 (not-expressed) or 1 (expressed) and is expressed as 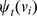. In a Boolean network, every gene expression profile at time 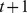 is completely determined by the expression profile of a set of genes 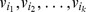 at time 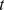 and the corresponding Boolean function 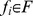. That is, we can write 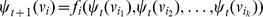.

**Table 3:** Count and probabilities table for 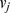, 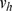 and 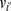 with misclassification error.

*v′_i_/v_j_v_h_*	00	01	10	11	*v′_i_/v_j_v_h_*	00	01	10	11
0	*n*_000_	*n*_010_	*n*_100_	*n*_110_	0	*r*_000_	*r*_010_	*r*_100_	*r*_110_
1	*n*_001_	*n*_011_	*n*_101_	*n*_111_	1	*r*_001_	*r*_011_	*r*_101_	*r*_111_

For convenience, we converted the Boolean network 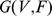 to the wiring diagram 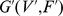 (See Figure 1) [37]. For each node 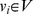, suppose 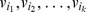 are the input nodes assigned to 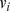. Then we construct an additional node 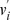 and connected the edge from 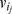 to 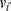 for each 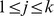. That is, the set of 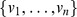 represents the gene expression profile at time 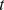 and the set of 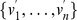 corresponds to the gene expression profile at time 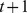. Hence we can treat the set of 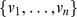 as the input values and the set of 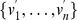 as the corresponding output values. Therefore, the output values of 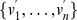 are determined by 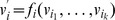.

### The Structure of Time Delay Boolean Network

In the previous subsection, we found that given the values of the node (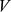) at time 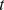, the expressions at time 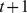 will be updated immediately by specific Boolean function (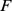). That is, for every gene 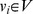, if the expression profile of a set of genes 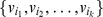 at time 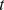 and the corresponding Boolean function 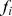 is observed, the gene expression of 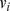 at time 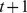 is determined by 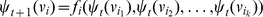. However, in real genetic regulatory situations, the deterministic system has been criticized due to the existence of misclassification error and noise. In addition, some of the gene expression may result in time delay when the gene is influenced by one or several input genes. That is, the induction of Boolean function may not activate the target gene immediately, but in the future. Hence, it would have been much more flexible to use a non-deterministic network system. In this subsection, we will consider two relationships between the Boolean function and the target gene instead of the deterministic relation.

**Table 4:** Splitting counts caused by misclassification error.

*v′_i_/v_j_v_h_*	00		01		10		11	
	*m*_000,000_	*m*_000,001_	*m*_010,000_	*m*_010,001_	*m*_100,000_	*m*_100,001_	*m*_110,000_	*m*_110,001_
0	*m*_000,010_	*m*_000,011_	*m*_010,010_	*m*_010,011_	*m*_100,010_	*m*_100,011_	*m*_110,010_	*m*_110,011_
	*m*_000,100_	*m*_000,101_	*m*_010,100_	*m*_010,101_	*m*_100,100_	*m*_100,101_	*m*_110,100_	*m*_110,101_
	*m*_000,110_	*m*_000,111_	*m*_010,110_	*m*_010,111_	*m*_100,110_	*m*_100,111_	*m*_110,110_	*m*_110,111_
	*m*_001,000_	*m*_001,001_	*m*_011,000_	*m*_011,001_	*m*_101,000_	*m*_101,001_	*m*_111,000_	*m*_111,001_
1	*m*_001,010_	*m*_001,011_	*m*_011,010_	*m*_011,011_	*m*_101,010_	*m*_101,011_	*m*_111,010_	*m*_111,011_
	*m*_001,100_	*m*_001,101_	*m*_011,100_	*m*_011,101_	*m*_101,100_	*m*_101,101_	*m*_111,100_	*m*_111,101_
	*m*_001,110_	*m*_001,111_	*m*_011,110_	*m*_011,111_	*m*_101,110_	*m*_101,111_	*m*_111,110_	*m*_111,111_

**Table 5:** The eight basic relationships and their probabilistic hypotheses and 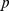-scores.

Relation	Hypothesis	Scores
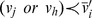	q_000_ = 0	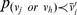
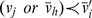	q_010_ = 0	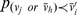
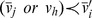	q_100_ = 0	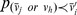
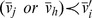	q_110_ = 0	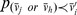
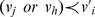	q_001_ = 0	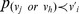
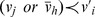	q_011_ = 0	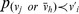
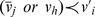	q_101_ = 0	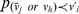
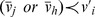	q_111_ = 0	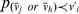

First, we will introduce the structure of time delay Boolean networks. Suppose there are 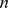 elements, 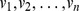 in a Boolean network. For any elements 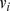 with specific Boolean function 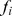, we have two kinds of pair wise relationship: prerequisite and similarity. We say that a Boolean function 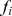 with specific 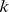 input genes 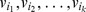 at time 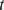 is the prerequisite for the target gene 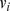 at time 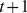, if the on-status of Boolean function at time t is necessary for the on-status of gene 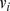 at time 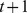. This relationship is denoted by 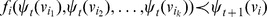. In other words, if the Boolean function 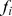 is not active at time 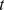, gene 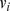 will be inactive at time 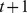. If it does not cause confusion, we will omit the notation of 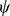 and input genes as denoted by 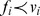. Moreover, for every gene 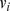, we use 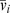 as its dual (from 0 to 1, or from 1 to 0) in this paper. Therefore, for any Boolean function and target gene with a prerequisite relationship there are a total of two possible relationships: 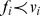 and 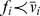. In this model, we do not consider the situation of a dual of Boolean function prerequisite to the target gene, that is 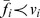 and 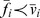. Since for any Boolean function whose dual is a prerequisite to the target gene, there must exist another Boolean function that is a prerequisite to the target gene. For instance, if 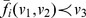, where 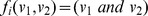, then 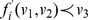, where 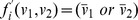. Therefore, for the prerequisite relationship, we only consider the Boolean function that is a prerequisite to target gene and the dual of target gene.

The other type of relationship between Boolean function and target gene is similarity. We say that the Boolean function 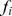 and target gene 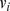 are similar if the status of the Boolean function and the status of the target gene are in the same expression, and we denoted this by *f_i_∼v_i_*. In the same way, we do not consider the situation of Boolean function similar to the dual of target gene such as *f_i_∼
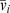
* in this study. Since if there is one Boolean function that is similar to the dual of target gene, there must exist another Boolean function that is similar to the target gene.

In the diagram, if a Boolean function 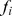 is a prerequisite to 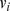, we draw a directed arrow from the vertex 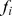 to 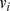 and if 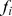 is similar to 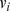, we use an undirected line to connect 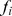 and 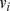.

**Table 6:** By the time delay Boolean network in Figure 1, we generate 100 samples with p = 0.05.

Samples		Hypotheses								Relation
Input Output		q_000_ = 0	q_010_ = 0	q_100_ = 0	q_110_ = 0	q_001_ = 0	q_011_ = 0	q_101_ = 0	q_111_ = 0	
*v*_1_,*v*_2_	*v*_1_^′^	0.493	0.418	0.273	0.379	0.148	0.178	0.372	0.343	
*v*_1_,*v*_3_	*v*_1_^′^	0.438	0.147	0.248	0.222	0.016	0.245	0.182	0.241	(*v*_1_ *or v*_3_) 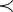 *v′*_1_
*v*_2_,*v*_3_	*v*_1_^′^	0.318	0.260	0.571	0.214	0.189	0.293	0.138	0.374	
*v*_1_,*v*_2_	*v*_2_^′^	0.326	0.300	0.304	0.297	0.091	0.092	0.232	0.209	
*v*_1_,*v*_3_	*v*_2_^′^	0.338	0.216	0.349	0.197	0.039	0.069	0.038	0.243	(*v*_1_ *and v*_3_) 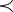 *v′_2_*
*v*_2_,*v*_3_	*v*_2_^′^	0.326	0.253	0.390	0.174	0.052	0.141	0.017	0.169	
*v*_1_,*v*_2_	*v*_3_^′^	0.211	0.011	0.355	0.029	0.040	0.228	0.011	0.294	
*v*_1_,*v*_3_	*v*_3_^′^	0.338	0.290	0.402	0.734	0.669	0.291	0.379	0.360	*v*_2_∼*v′_3_*
*v*_2_,*v*_3_	*v*_3_^′^	0.247	0.312	0.030	0.011	0.039	0.011	0.283	0.241	

In the model of time delay Boolean network we proposed, the output of the gene expression is not completely determined by the input state and Boolean function. The output expression may have more than one possible result in the time delay Boolean network. We illustrate the above construction by an example in Figure 2. It has three elements, one similarity and two prerequisite relationships. The possible outputs for every input state are listed in the right part of the graph. If we knew the network structure, some of the inputs would have more than one possible output expression in the time delay Boolean network.

**Figure 3:**
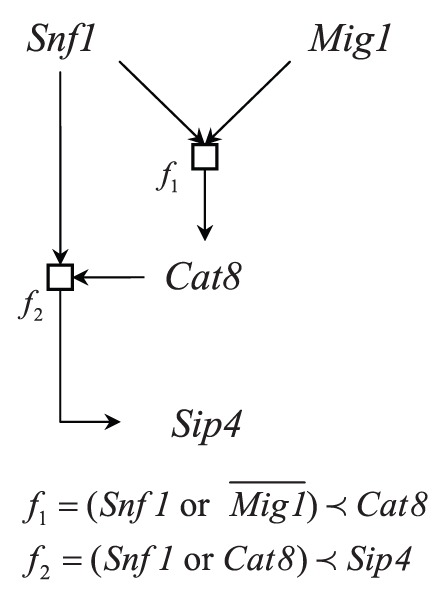
Network reconstruct from the expression data of yeast Saccharomyces cerevisiae.

## Methods

### Identification Algorithm

First, we only consider Boolean networks in which the maximum number of input genes is bounded by a constant 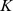 for every target gene, because it has been proven that the number of profiles required grows exponentially if 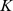 is not bounded [38]. For simplicity, we only show algorithms for the case of 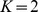. However, the algorithm can be intuitively generalized to any 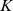 in a straightforward way. For the inference of genetic network, we need to clarify the following questions for each target gene.

- Which input genes will affect the target gene?
- What kind of Boolean functions will be used for combining those input genes?
- What kind of relationship exists between the Boolean function and the target gene?

In this subsection, we propose an algorithm to clarify the above questions. The algorithm below is conceptually very simple since it simply uses output Boolean functions with input genes and relationships with target genes that are consistent with the data. First, for each output gene expression at time 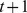 such as 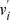, we consider all the pairs of elements in 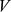 at time 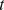, for instance 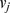 and 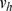. Then we count the eight incidents of (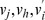) being (0,0,0), (0,0,1), 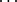, (1,1,1) from the sample and arrange them in a 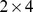 table; see the left part of Table 1. We mark a cell “+” if the count is positive and mark it “0” otherwise.

For detecting whether there exists a Boolean function which is a prerequisite to the target gene, we will compare the 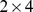 output table with the left four basic relationships in Table 2. We consider the basic relationships to be consistent with the output table if the position of 0 cell in the basic relationships is also 0 in the output table. By comparing the output table with the four basic relationships, we can find relationships that are consistent with the output tables. If there is more than one relationship that is consistent with the output tables, we would use the Boolean logic gate “and” to combine the Boolean function and transfer the result to another Boolean function. Hence, the final Boolean function is the prerequisite to the target gene. Similarly, by comparing the 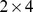 output table with the right four basic relations in Table 2, we could get another Boolean function which is the prerequisite to the dual of target gene.

Moreover, if only one Boolean function occurred in above relationship, that is, if there is only one Boolean function that is the prerequisite to the target gene or the dual of target gene, we will treat that relationship as our final relationship between the Boolean function and the target gene. However, if both of the two prerequisite relationships happened (i.e. 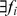 and 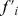 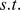 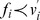 and 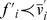), we need to check whether these two relationships are in conflict. If the dual of 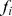 is equivalent to 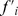, our conclusion for inference will be that 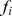 is similar to the target gene (that is, 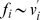); otherwise, we will treat it as if there is no relationship between the input genes and the target gene because we did not gather enough information to judge true relationships between 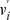 and (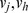) at this moment. By the above identification procedure, we can find the corresponding input genes, Boolean function and their relationship for every target gene.

### Identification Algorithm with Noisy Array

In previous subsection, we discussed an identification method for data without noise. In this section we will consider the situation of noisy array data. We assume that every element in the entry of (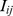, 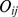), 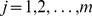 switches to its reverse status with a misclassification probability 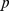 independently; that is
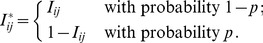
(1)

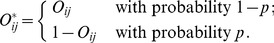
(2)


Thus, the observed array (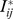, 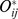) contains misclassification error. Our goal is to reconstruct time delay Boolean network from noisy array of binary data (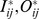).

Similar to section 2, we assume that the maximum number of input genes is bounded by 2 for every target gene. We treat the data in the 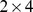 table as a multinomial distribution with eight cells whose probabilities are 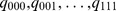 as shown in the right part of Table 1, where 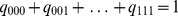. Similarly, we extract the data with misclassification error for every output gene and each pair of input genes as the 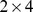 table. Now the observed data 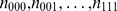 are not generated from the multinomial 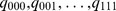, but from another multinomial 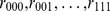 as shown in Table 3, where 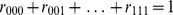.

Because of the misclassification error, a portion of the samples of 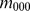 may change to the other seven cells. We use the notations of 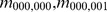, 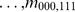 to represent the counts of eight cells changed from 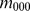. Analogous notations are defined for 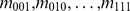. The splitting is shown in Table 4. Consequently, the generated probabilities (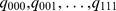) are calculated as follows: 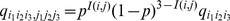, where 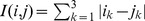. Here, we adopt the notation 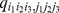 analogous to 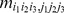. The above parameters and splits are shown in Table 4. In the table, it is easy to find that the correspondence between two sets of counts and probabilities is the following:
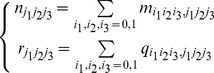


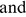
(3)

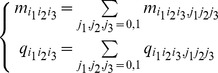



For the complete data 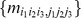, the log-likelihood is given by
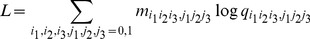
(4)where 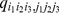 are those splitting probabilities. Since the complete data 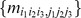 are not observable, we use the EM algorithm to maximize the log-likelihood. In the E-step, the splitting counts of complete data 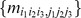 are evaluated by the conditional expectations using the current values of the parameters by the following formula
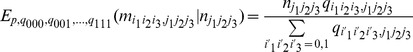
(5)where 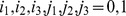. One probabilities of 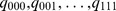 are zero in those different hypotheses specified in Table 5. In the M-step, we maximize the conditional expectation of the log-likelihood for the complete data to obtain the maximum likelihood estimates (MLEs) of the parameters. According to the MLEs, we can compute the 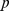-score for every pair of input genes and target gene, which are obtained by estimating for the misclassification probability under every prerequisite relationship.

For the first step, we would like to determine the most probable relationships between every pair of input genes and one output gene. Next, we find the most probable Boolean function with pair input genes for every output gene and select candidate pairs of input genes and output gene for the watch list. Finally, we reconstruct a time delay Boolean network by integrating the relationship of those genes selected.

For one output gene 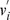 and a pair of input genes 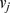 and 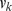, we define the 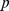-scores 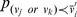, 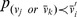, 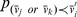, 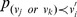, 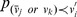, 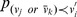, 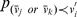 are, respectively, the maximum likelihood estimates of p under the triangular model: 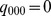, 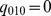, 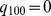, 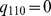, 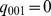, 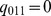, 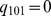, 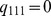.

According to the EM algorithm described above, we can evaluate the 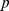-score for every output gene. We use the MLE 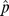 to measure how well each hypothesis fits: the smaller the score is, the more likely that the corresponding hypothesis could be true.

If the samples are generated from a time delay Boolean network, 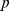-score are quite useful for the discovery of true relationships. Here we can consider the *maximum compatibility criterion*: to choose the maximum threshold value so that the selected relationships contain no conflicts [34]. We collect those relationships whose 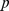-scores are smaller than a threshold. Known biological results are helpful for the determination of a threshold. For example, if we know the relationship 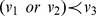 is true, then the 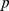-scores smaller than 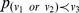 should be in our watch list. As more relationships are included in the watch list, the more likely we are to observe incompatible ones. In general, we can choose the threshold that allows the maximum number of relationships with no conflicting relationships. Next we will demonstrate the method by illustration examples.

## Results and Discussion

### Theoretical Results

First, we will analyze the number of input/output pairs required for the network reconstruction of time delay Boolean network to be unique. The theoretical results of classical Boolean networks only consider the similar relationship [27], [38], [39]. The following results prove the theoretical results time delay Boolean networks that has a more flexible structure and consider both similar and prerequisite relationship.

#### Proposition 1

*For all subsets of* 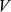 *with* 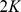 *genes,* *if all assignments (i.e.,* 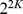 *assignments)* *of Boolean values appear in input expression patterns and all of its possible output expression patterns of the target gene are present, the identification of genetic network is determined to be unique, if it exists.*

(Proof) Let 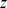 be any gene in 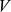 and suppose 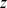 is controlled by a Boolean function 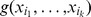 with similarity or prerequisite relationship (i.e., 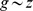 or 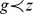). If the Boolean function 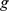 is similar to 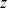, the case is proved for the classical Boolean networks in Akutsu et al. (1998). Next, we consider the case of Boolean function 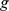 as a prerequisite to 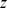. In this case, there must exist a specific input value 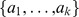 for 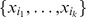 such that 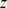 have two possible values 0 and 1. Hence, any other genes would not control 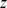 because all assignments of Boolean values are appearance. Let us illustrate the above statement by the example for the case of 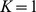 and 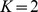. If 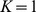 and 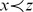, when the input of 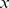 is 1, the outcome of 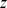 being both 0 and 1 will appearance. Therefore, given the input of 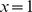, the outcome of 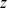 is not deterministic no matter the value of any other gene 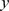 is 1 or 0. Hence, any other gene 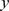 would not affect gene 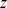. If 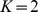 and 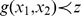 for some Boolean function 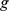, there must exist an input 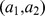 such that 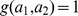. Then, for any other pair of gene 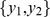 where 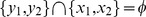, the outcome of 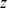 is not deterministic for any input of 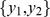, if the input of 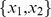 is 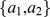. In a case of 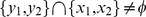, we can prove that gene 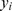 which does not belong to 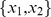 would not affect the gene 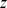 in a similar way.

#### Proposition 2

*The probability that one sub-assignment with all of its possible results in the target gene does not appear among m random input expression pattern is at most* 
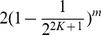
.

(Proof) For any fixed set of nodes 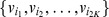, the probability that a sub-assignment 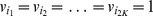 does not appear in one random input expression pattern is 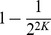. Thus, among the 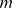 random input expressions, the probability that 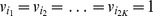 appears is 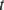 times is equal to 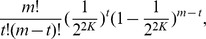 where 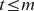. In addition, the probability that all of the possible results in the target gene does not appear among 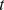 times input is smaller than 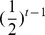 for 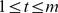 and equal to 1 for 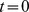. Hence the probability that one sub-assignment and all of its possible results does not appear among 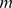 random input expression is smaller than 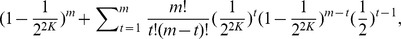 and this can be bounded by 
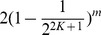
 by an algebra calculation.

Next we prove the main theorem.

#### Theorem 1

*For the identification of one time delay Boolean network of n nodes with maximum indegree* 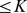, 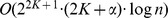 *uniformly and randomly sampled input patterns are sufficient for exact inference with probability at least* 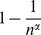.

(Proof) We consider the probability that the condition of Proposition 1 is not satisfied under 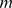 random input expression patterns.

By Proposition 2, the probability that 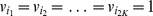 with all of its possible results in the target gene does not appear among the 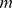 random input expression patterns is bounded by 
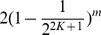
 for any fixed set of nodes 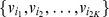. Since the number of combinations of 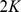 nodes from a set of 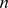 possibilities is bounded by 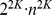, the probability that the condition of Proposition 1 is not satisfied is at most 
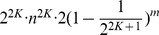
. It is not difficult to see that 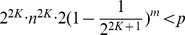 holds for 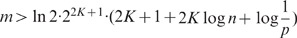. Hence, we obtain the theorem by letting the non-identification probability 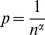.

Next we develop an information theoretic lower bound on the number of input/output pairs needed for the identification of a time delay Boolean network.

#### Theorem 2

*If the maximum indegree* 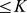, *at least* 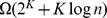 *input/output pairs are required for the identification of a time delay Boolean network in the worst case.*

(Proof) The number of time delay Boolean networks is given by all the possible combination of Boolean function with 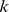 nodes from a set of 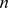 possibilities with all possible relations between Boolean functions with target node. Since there are 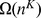 possible combinations of input nodes, 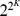 possible Boolean functions and 3 possible relations between Boolean function with each node, there are 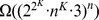 Boolean networks whose maximum indegree is at most 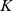. On the other hand, there are at most 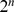 possible output patterns with one input expression pattern. Therefore, 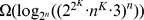 which is the same as 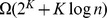 input/output pairs are required in the worst case.

### Example with Simulation and Real Data

We will illustrate our method by the example described in Figure 2. For the pair of samples consist of three elements list in the right part of Figure 2, we uniformly generated 100 input samples and their corresponding possible output samples with misclassification probability 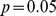. For the prerequisite relationship, if the status of Boolean function with input genes is on, then we allow the output value to have equal probability of on or off. The data can be arranged as input/output sample similar to that obtained from the microarray data with time. Namely, the input of each sample can represent the gene expression at time 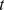 and the output can represent the gene expression at time 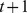. For each pair of input and output genes, we compute the 8 basic 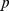-scores that represent the 8 basic hypotheses in Table 5 for all of pair input genes and output genes. After the calculation, the simulation results of every 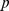-score are listed in Table 6.

Beside the example with 3 elements, in order to shows the superiority of the proposed method can be applied to a larger network, a more comprehensive example with a larger network is given in Figure S1.

Next, we have to decide the threshold for choosing the relations. When we increase the threshold of the 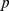-score, the relations whose 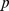-score are smaller than the threshold will be chosen. Moreover, when the number is 0.138, the conflict occurs, since we have 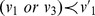 and 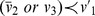. However, in our model, there are at most two genes that would affect an output gene. Therefore, we can choose 0.138 as our threshold and include relations whose 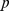-score is smaller than the threshold. By these procedures, we can reconstruct the time delay Boolean network identical to Figure 2.

In the area of gene regulatory network study, Schuller has summarized regulatory cis-acting elements of structural genes of the nonfermentative metabolism and described the molecular interactions among general regulators and pathway-specific factors [40]. In the gene regulation of gluconeogenesis by Sip4 and Cat8 pathway, the carbon source control could be identified for the regulator Cat8; see (Figure 6) in Schuller [40]. In this study, we apply our proposed approach to explore the expression profiles and show some exploratory result on the Cat8 pathway.

In order to demonstrate the effectiveness of reconstruction, we use the microarray expression dataset of yeast *Saccharomyces cerevisiae* produced by DeRisi et al. [1] and Spellman et al. [41]. In total, the data is comprised of 41 experiments after filtering out experiments with missing values. By these experimental microarray data sets, we can use our proposed method to reconstruct the biological pathway and the genetic regulation network result is shown in Figure 3. The result is consistent with the genetic network in the literature. That is, the restraint of Mig1 or activation of Snf1 is a prerequisite for the decreasing of Cat8. Moreover, the restraint of Snf1 or Cat8 is a prerequisite for the decreasing of Mls1. However, the negative similarity between Snf1 and Mig1 is undetectable in our current model.

### Conclusions

In this paper, we have introduced the model of time delay Boolean network that generalizes the Boolean network model in order to cope with dependencies that have two kinds of relationships: similarity and prerequisite. The approach for reconstruction of genetic network inference from gene expression data relies on the assumption that the expression of a gene is likely to be controlled by a relatively small number (say 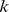) of genes. Also, some bounds on the size of data required for the identification of the time delay Boolean networks under constant of indegree are stated and discussed. Moreover, the algorithm of the network reconstruction from noisy array data is developed.

One characteristic of a Boolean network is that all the variables in the graph are binary. If the data we observed is continuous or quantized to have more than two levels, we need to discretize them. For microarray data, the ratios of expression level would be one possible approach of discretization. That is, we can treat the gene as on (active) if the log-ratio of its expression is larger than zero. We treat it as off (inactive) otherwise. In general, biological background knowledge will be helpful for setting thresholds for discretizaion. On the other hand, if the samples are obtained from a time course, then we can consider the gene as on or off by detecting whether the gene is either increasing or decreasing with time.

The work in progress is aimed at evaluating the effectiveness of the described approach for inferring genetic networks from biological gene expression time series data. Besides that, implementation on some other real biological data is also an important task.

For the implement of the network reconstruction algorithm, the greatest complexity is the computation of 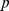-score for each of the 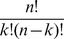 input elements and 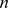 output elements, where 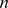 is the number of elements and 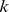 is the number of indegree. It is an iterative algorithm to compute the MLE for the 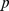-scores by EM procedure while the common practice is to set an upper bound for iterations in numerical implementation. Consequently, this keeps the 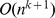 complexity for the computation of MLE. In addition, the sorting algorithm for the 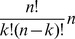 data cost 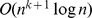 in terms of time. Hence, the overall time complexity for the network reconstruction is 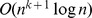 for this algorithm.

## Supporting Information

Figure S1 **An example of genetic network with 8 nodes.**
(PDF)
