# Supplementary material for: Inference of Biological Pathway from Gene Expression Profiles by Time Delay Boolean Networks
Source: PLoS One. 2012 Aug 31;7(8):e42095. doi: 10.1371/journal.pone.0042095 (PMC3432056; doi:10.1371/journal.pone.0042095)
Supplement: Figure S1 — An example of genetic network with 8 nodes. (PDF) [file pone.0042095.s001.pdf]

## Supporting Information

# A genetic network with 8 elements

In order to show that the proposed method can be used by a larger network, we construct a larger genetic network as shown in Figure S1.

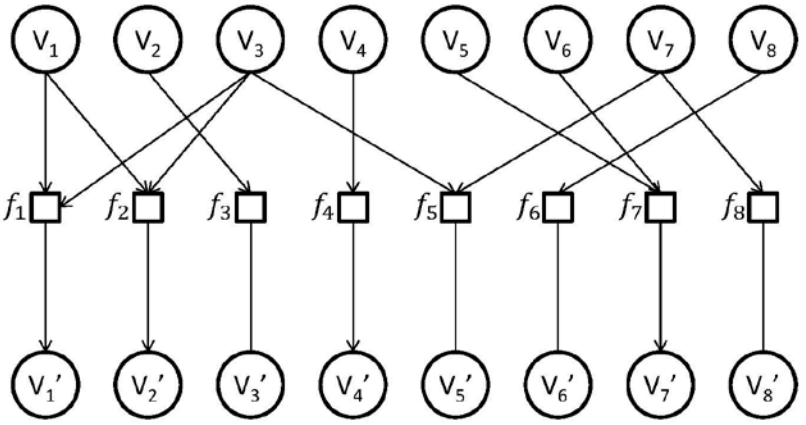


$$f_1 = (v_1 \text{ or } v_3) \prec v_1'$$
$$f_2 = (v_1 \text{ and } v_3) \prec v_2'$$
$$f_3 = (v_2) \sim v_3'$$
$$f_4 = (v_4) \prec v_4'$$
$$f_5 = (v_3 \text{ or } v_7) \sim v_5'$$
$$f_6 = (v_8) \sim v_6'$$
$$f_7 = (v_5 \text{ or } v_6) \prec v_7'$$
$$f_8 = (v_7) \sim v_8'$$

Figure S1. An example of genetic network with 8 nodes.

According to the relationship of Figure S1, we uniformly generated 1000 input samples and their corresponding possible output samples with misclassification probability $p$=0.05. Then, we used the proposed method as described in Section 3 to reconstruct our genetic network. The result shows that we can reconstruct the original network of Figure S1 with no error. However, if we decrease the sample size by using only 100 input samples, the relationship of $(v_1 \text{ and } v_3) \prec v_2'$ will be treated as $v_1 \prec v_2'$.

In addition, we conduct the comparison study with the directed acyclic Boolean network approach proposed by Li and Lu (2005) for this case. The result shows that the relationship of $(v_1 \text{ and } v_3) \prec v_2'$ and $(v_1 \text{ and } v_3) \prec v_2'$ cannot be detected by the directed acyclic Boolean network approach for sample size of 1000. Similarly, the relationship of $(v_1 \text{ and } v_3) \prec v_2'$ also have been treated as $v_1 \prec v_2'$ for sample size

of 100. The performances for the two reconstruction approaches with different simulated sample sizes are listed in Table S.1.

Table S.1. The accuracy rates for different reconstruction approaches with different simulation sample sizes.

| | Simulation sample size | |
|---|---|---|
| Reconstruction approach | 100 | 1000 |
| Time delay Boolean network | 87.5% | 100% |
| Directed acyclic Boolean network | 62.5% | 75% |
